# ‘They’re waiting for us to break before they listen’: healthcare workers’ perspectives on how to mitigate moral distress in British Columbia, Canada

**DOI:** 10.1017/S1463423626101352

**Published:** 2026-06-19

**Authors:** Muhammad Haaris Tiwana, Alice Murage, Jorges Andres Delgado-Ron, Rosemary Morgan, Julia Smith

**Affiliations:** 1 Faculty of Health Sciences, Simon Fraser University, Canada; 2 Johns Hopkins Bloomberg School of Public Health: Johns Hopkins University, USA; 3 College of Health Sciences, VinUniversity, Hanoi, Vietnam

**Keywords:** gender, healthcare providers, moral distress, qualitative methodology, race

## Abstract

**Objectives:**

Moral distress among healthcare workers can have serious repercussions, including reduced morale, decreased quality of care, apathy, burnout, and workforce attrition. While much research has documented moral distress, few studies have considered how to effectively mitigate it. This study sought to enhance understanding of moral distress by exploring not only who is affected and why, but also examining strategies for addressing it.

**Methods:**

An online survey was conducted with primary care healthcare professionals in British Columbia, Canada, from October to December 2022, including physicians, nurses, homecare aides, and long-term care aides. The survey gathered sociodemographic data, documented experiences of moral distress, and explored methods used to mitigate it. We specifically analysed responses to two open-ended questions on how respondents addressed moral distress, categorizing responses by profession, gender, and racial identity.

**Results:**

Employing a socio-ecological framework, we organized mitigation strategies across four levels: individual, interpersonal, community/organizational, and societal/systemic. Strategies ranged from personal coping mechanisms – such as mindfulness and exercise – to more collective actions aimed at changing workplace culture and policies. At the organizational and systemic levels, participants offered recommendations for structural reforms to improve support for healthcare staff.

**Conclusion:**

This study highlights the multifactorial nature of moral distress, showing that it arises from a complex interplay of systemic, organizational, interpersonal, and individual factors. Our findings emphasize the importance of developing multi-level approaches to mitigate moral distress in healthcare, addressing both immediate individual needs and broader organizational and policy changes that promote a healthier work environment.

## Introduction

Moral distress, defined as ‘a phenomenon in which one knows the ethically right action to take but is systemically constrained from taking it’, has developed as a concept to understand the unique psychological and emotional response of health and social care workers (Berhie *et al*., 2020). Responses associated with moral distress include feelings of powerlessness, self-blame, anger, frustration, discouragement, anxiety, and depression (Jameton, 2017; Epstein *et al*., 2019; Dodek *et al*., 2021). These effects, in turn, can contribute to the deterioration of morale and decrease in quality of care, as well as apathy, burnout, and attrition. For instance, Dodek *et al*. (2021) reported that one-third of clinical care physicians surveyed in Canada had considered leaving their positions due to moral distress. Similarly, Webber *et al*. (2022) highlighted how human resource shortages and systemic challenges drive moral distress among Canadian community healthcare providers.

Such findings demonstrate the importance of addressing moral distress not only to mitigate attrition in the healthcare sector but also to support the mental health of workers and ensure provision of quality care to patients. For instance, moral distress contributes to higher staff turnover, which disrupts continuity of care, increases risk of adverse events, and lowers patient satisfaction (Aiken *et al*., 2012; Garcia *et al*., 2019). Moral distress can also lead to burnout, which in turn is associated with apathy and compassion fatigues as well as increased risk of making medical errors (Cherny *et al*., 2015). However, while there is substantial research on experiences and harms of moral distress in general, little of this goes beyond documenting moral distress to consider how to mitigate it (Forozeiya *et al.*, 2019). What research does exist has focused on individual coping strategies, even though moral distress is, by definition, a response to systemic conditions beyond one’s control (Booi *et al*., 2021). There is a need to not only understand who is affected by moral distress, how, and why, but also to conceptualize strategies to mitigate it.

This paper contributes to filling this gap by reporting on qualitative data collected through a moral distress survey of healthcare workers (doctors, nurses, long-term care [LTC], and in-home or community care providers) in British Columbia (BC), Canada. We approach this analysis through the socio-ecological framework – a model that has been extensively applied across various aspects of the healthcare sector to understand the diverse and contextual factors affecting healthcare providers (Litchfield *et al*., 2021; Caperon *et al*., 2022). The framework includes four levels of analysis (systemic/societal, community/organizational, interpersonal, and individual) to identify determinants and contextual influences related to moral distress. It further acknowledges the complex interactions that occur among and within each level. As sociocultural, political, and economic drivers structure the healthcare environment, this holistic approach provides insights into the emotional and psychological responses of healthcare providers (Davidson *et al*., 2018), as well as offers pathways to mitigating the impacts of moral distress and other ethical dilemmas they may face (Davidson *et al*., 2018). The socio-ecological framework is also useful in elucidating structural inequities such as racism and sexism as it aids in highlighting intersecting associations across multiple systems (Lanier and DeMarco, 2015). Consideration of how these dynamics interact with moral distress is crucial considering that most healthcare workers in Canada are women (over 80%), and an increasing portion is racialized (StatsCan 2020; CIHI, 2023).

## Methods

### Study instrument and data analysis

We conducted an online survey with physicians, nurses, homecare aides, and LTC aides employed in British Columbia (Canada) between October and December 2022. We used convenience sampling for the survey. English-speaking physicians, nurses, homecare aids, and LTC aids were eligible if currently employed in BC at the time of the survey. Participants were recruited through email and social media via primary care healthcare worker unions such as the Hospital Employees Union and occupational organizations, such as SafeCare BC. Data collected included sociodemographic data, experiences of moral distress, and strategies applied to mitigate moral distress. Respondents were allowed to skip questions to mitigate forced recollection of uncomfortable or distressing experiences. Details about the study and the quantitative results detailing the degree and determinants of moral distress have been reported elsewhere (Smith *et al*., 2023; Delgado-Ron *et al*., 2024; Smith *et al*., 2024). Among the 2,918 respondents who met the inclusion criteria and had valid responses (survey completion ≥ 3 minutes), 734 answered either or both of two open-ended questions which allowed us to explore how healthcare workers mitigated moral distress: *‘Are there any coping strategies you prefer or that you would like to mention and elaborate on?*’ and *‘Is there anything else you would like to share about moral distress?’* These participants constituted our analytic sample for this paper. We extracted the text responses to these questions along with a subset of sociodemographic characteristics of the respondents (profession among physicians, nurses or homecare and LTC aides, gender, and if they identified as racialized. In this study, the term racialized is used to refer to individuals from ethnic minority backgrounds who experience social and structural processes of racialization.

The data were coded using a mixed inductive and deductive approach. We adapted the four-level socio-ecological model to guide the thematic analysis deductively (Wu *et al.,* 2022). Then, we employed content analysis, a research method involving the subjective interpretation of text data through systematic coding and theme identification (Popping, 2015; Hsieh & Shannon, 2005), to explore strategies for mitigating moral distress from the structural to the individual level. Using the levels of analysis within the socio-ecological model as top-level themes, two authors conducted a close reading of the data and identified further codes inductively, which were organized as subthemes through discussion with the full author team. Once the themes and subthemes were established, one author coded the data independently using NVivo software, frequently discussing classifications and sharing the decision-making process with the co-authors. We maintained an audit trail of coding decisions, documented coder reflections on positionality and potential bias. We also reviewed themes against raw data to validate fit and coherence. A spreadsheet database was created to code and group the thematic findings.

### Ethics

This study was approved by the Simon Fraser University (SFU) Research Ethics Board (30001218) and consistent with the principles of the 1964 Helsinki Declaration and its later amendments. The survey participants were informed about the research objective and were made aware that participation was voluntary and that they could withdraw from the survey at any time.

## Results

### Sample characteristics

Among the 734 respondents (Table 1) included in our analysis, a majority were LTC aides (81%), followed by nurses (14%) and physicians (4%). Most respondents were women (84%) and racialized (87%).

**Table 1.** Characteristics of respondents (*N* =734) Table 1 long description.

Profession	Total respondents (*N*) = 734		
	Gender		Racialized persons
Long-term care provider (*n* = 598)	Man	37 *(6%)*	19 *(51%)*
	Woman	532 *(89%)*	489 *(92%)*
	Gender minority	29 *(5%)*	14 *(48%)*
Physician (*n* = 30)	Man	9 *(30%)*	7 *(77%)*
	Woman	10 *(33%)*	10 *(100%)*
	Gender minority	11 *(36%)*	9 (*82%)*
Nurses (*n* = 106)	Man	15 *(14%)*	12 *(80%)*
	Woman	75 *(71%)*	68 *(91%)*
	Gender minority	16 *(15%)*	12 *(75%)*

To better understand how healthcare workers navigate moral distress, we applied the socio-ecological framework to categorize our findings into subthemes across different levels: individual, interpersonal, community/organizational, and societal/systemic (Table 2). By mapping respondents’ strategies for mitigating moral distress to these levels, we could identify both the actions taken by individuals and the broader systemic factors influencing their experiences. The strategies employed ranged from personal coping mechanisms to more structural interventions aimed at addressing workplace culture and policies. Within the organizational and systemic levels, the participants shared aspirational mitigation strategies/recommendations.

**Table 2.** Socio-ecological framework and thematic analysis Table 2 long description.

Themes/socio-ecological levels	Subthemes from our data	Suggested strategies
Individual behaviours, knowledge, attitudes, capabilities, etc.	Creative and leisure activities	Protected time outside of work
	Therapeutic practices	Investments in mindfulness training, therapy and other mental health supports
Interpersonal Formal and informal networks and support systems, relationships between individuals	Collegial Relationships	Peer support opportunities Regular team meetings
	Family and Friends	Foster and strengthen personal connections
Community/organizational formal and informal regulations, built environment, relationships between organizations/networks	Workplace Safety	Proactive, instead of reactive, approach Occupational health teams
	Regulations and Bureaucracy	Streamline processes Flexibility and rapid decision-making Engagement of frontline staff in policymaking
	Administrative Support	Improved communication between frontline staff and management Mentorship programmes
Societal/systemic Laws and policies including those related to resource allocation; social norms and values	Addressing racism	Mandatory trainings Safe spaces to address concerns Institutional policies on racial equity Commitments from leadership
	Staffing shortages and retention	Increased investments Improved financial oversight and transparency
	Resources for rural health	Policies and resources specific for rural communities Investments in staffing and virtual care technology

### Individual level

Respondents highlighted various personal strategies they employed to cope with the moral distress they experienced in their roles. These strategies were deeply personal and varied widely (e.g., crying, debriefing, journaling). Many emphasized the importance of engaging in activities that brought them joy, relaxation, or a sense of control. One common coping strategy was engaging in creative and leisure activities such as singing, music therapy, painting, yoga, or gardening. These activities provided an outlet for emotional expression and a way to decompress from daily stress. For instance, one respondent shared, ‘I usually like singing. When I am in a bad mood, I will go to sing loudly. It’s a very special decompression for me’ (*LTC, racialized woman* Participant Identification (PID) 3271). Physical activity was frequently mentioned as well with some respondents noting that exercise, whether running, walking, or practising sports, played a critical role in their mental and physical well-being. ‘Exercise is my go-to strategy. It helps me clear my mind and recharges me after a tough day at work’ (*LTC, white woman* PID 3984). However, others like this nurse said, ‘I think many nurses in acute care like me also find it difficult to use exercise as a form of stress relief because work is so physically demanding. My body is in too much pain after a shift to exercise, sometimes for more than a day (and I am only in my 20s)’ *(racialized woman* PID 1161).

Additionally, the use of therapeutic practices such as mindfulness, meditation, and attending therapy sessions was viewed as essential tools for processing emotions, managing anxiety, and maintaining mental health. ‘Music therapy has been a lifesaver for me. It helps me process my emotions and gives me a break from the constant stress’ (*LTC, racialized gender minority* PID 3568). This suggests that therapeutic activities can be both preventive and remedial, providing individuals with strategies to manage their stress before it becomes overwhelming.

### Interpersonal level

#### Collegial relationships

Positive interpersonal relationships at work were noted to be a protective factor against moral distress. Particularly, that supportive colleagues can empathize with their challenges and offer encouragement during difficult times. These relationships were described as vital for maintaining morale and fostering a sense of belonging within the workplace. One participant noted, ‘Having a forum where we can share our experiences and support each other can make a big difference. It’s important to know that we are not alone, and we are collectively taking steps to address these issues’ (*LTC, racialized woman* PID 2023). Respondents noted the value of peer support in creating a more resilient workforce where individuals feel connected and supported. In addition, respondents highlighted the importance of structured opportunities for team-building and open communication. Regular team meetings, peer-to-peer check-ins, and support groups were cited as effective ways to strengthen interpersonal bonds and create a more cohesive work environment. These interactions provided a platform for healthcare workers to express their concerns, share coping strategies, and collectively address challenges, thereby reducing the emotional burden of moral distress.

#### Family and friends

Social connections outside of work also emerged as a critical coping mechanism, with respondents emphasizing the importance of spending time with friends, family, and pets. These interactions provided emotional support, helping individuals feel connected and valued outside of their professional roles. One respondent explained, ‘Spending time with friends and family is my way of escaping the stress of work. It reminds me of what’s really important and helps me keep things in perspective’ (*LTC, racialized man* PID 4097). Social support was viewed as essential to coping with moral distress experiences at work, maintaining positive mental health, and reducing feelings of isolation.

### Organizational level

#### Workplace safety

Healthcare workers reported feeling unsafe due to inadequate protective measures, insufficient personal protective equipment, and exposure to hazardous working conditions. Unsafety at work contributed to their experiences of moral distress; they felt unable to offer care without compromising their health. As surveys were completed between October and December 2022, risk of COVID-19 infection was frequently referred to. Respondents highlighted that the lack of adequate safety protocols jeopardized not only their health but also the health of their patients and families. This dual concern – of potentially contracting and spreading illness – exacerbated moral distress, as healthcare workers were forced to navigate the ethical dilemma of balancing their duty to care for patients with the need to protect themselves and their loved ones. One respondent shared, ‘The fear of bringing the virus home to my family adds an extra layer of stress. It’s not just about me; it’s about everyone I come into contact with’ (*LTC, racialized woman* PID 2317). Respondents felt that institutional policies around workplace safety were reactive rather than proactive, often implemented only after significant pressure from workers or unions. This lag in response time contributed to a sense of frustration and mistrust in their institution’s ability to adequately protect its workforce.

To address these issues, respondents recommended that healthcare organizations adopt a more proactive approach to occupational health and safety. Additionally, they called for dedicated occupational health teams that work closely with staff to identify potential hazards and implement preventive measures. One participant suggested, ‘Having a dedicated team focused on occupational health would make a huge difference in how safe we feel at work. It’s about being prepared, not just reacting to crises’ (*Physician, racialized woman* PID 2459).

#### Regulations and bureaucracy

Respondents pointed to the complexity and rigidity of healthcare regulations, which often hindered their ability to provide timely and effective care, contributing to their moral distress experiences. Bureaucratic red tape, cumbersome approval processes, and inflexible policies were cited as barriers that slowed down decision-making and limited their autonomy and hence timely care to patients. For example, one respondent remarked, ‘There are so many hoops to jump through just to get approval for basic patient care. It’s frustrating when the system is more focused on paperwork than on actually helping patients’ (*LTC, racialized man* PID 3127). Organizational bureaucratic burdens were described as contributing to delays in care and exacerbating feelings of helplessness and frustration.

To address these challenges, respondents advocated for policy reforms that streamline administrative processes and reduce unnecessary regulatory barriers. They called for greater flexibility in institutional regulations, allowing for more rapid decision-making and adaptation to the evolving needs of patients and healthcare workers. Additionally, respondents recommended involvement of frontline workers in the policy-making process to ensure that regulations are grounded in the practical realities of patient care. One participant proposed, ‘Policy reform needs to be informed by those on the frontlines. We’re the ones who see how these regulations play out in real-time, and our input could help create more effective and responsive policies’ (*nurse, racialized man* PID 3278).

#### Administrative support

One of the most frequently cited drivers (71%) of moral distress among respondents at the organizational level was lack of support from healthcare administration. This supported existing literature highlighting the relationship between low level of perceived organizational support and high moral distress in hospitals (Robaee *et al*., 2018; Delgado-Ron *et al*., 2024). Respondents expressed frustration with leadership that seemed disconnected from the realities of frontline work. Many healthcare workers felt that their concerns were not being heard or adequately addressed by those in positions of power. This disconnect often resulted in a sense of abandonment, where staff felt left to manage overwhelming workloads and challenging situations without sufficient resources or guidance. For example, one respondent noted, ‘It often feels like the administration is in a different world – they set policies without understanding the realities on the ground. We are left to pick up the pieces when things go wrong, and there’s rarely any acknowledgment of the toll this takes on us’ (*LTC, racialized woman* PID 491). Another respondent echoed these concerns, stating, ‘When we voice our concerns about the current situation or the lack of necessary equipment, we’re often met with silence or vague promises of future changes that never come. It feels like they’re waiting for us to break before they listen’ (*LTC, racialized woman* PID 573). Another LTC worker expressed that, ‘The worst is knowing we could effect change, but suggestions are spoken into the wind. It is like screaming into a vacuum or banging your head against a wall’ (*LTC, racialized man* PID 1172).

Many respondents also reported feeling underappreciated and undervalued by their organizations, with little recognition for their hard work and dedication. One participant explained, ‘Lack of support from management and not feeling appreciated enough adds to the stress of an already demanding job’ (*LTC, racialized woman* PID 3342).

Respondents noted such challenges might be mitigated through improved communication between frontline workers and administration. They emphasized the need for leadership to be more attuned to the realities of frontline work and to involve healthcare workers in decision-making processes. A nurse noted, ‘If there was more transparency and two-way communication, we could solve problems together rather than just being told what the latest policy is, we need leaders who are willing to listen and act’ (*nurse, racialized woman* PID 820). Mentorship was also identified as a strategy to increase healthcare worker support.

Some respondents mitigated or coped with experiences of moral distress by raising concerns with, and seeking support from, their supervisors. They noted that effective supervision was characterized by open communication, regular feedback, and a genuine interest in the well-being of staff. One respondent mentioned, ‘Knowing that my supervisor has my back makes a huge difference. It’s reassuring to know that they’re there to listen and to support me when things get tough’ (*nurse, racialized man* PID 4097). Respondents further called for the implementation of formal mentorship programmes and professional development opportunities. One respondent emphasized, ‘Mentorship shouldn’t be an afterthought. It’s vital for retaining staff and helping us cope with the emotional toll of the job. A mentor who has been through similar experiences can provide both guidance and reassurance that you’re not alone’ (*LTC, racialized woman* PID 945).

### Societal/systemic level

#### Improving staffing and retention

The issue of understaffing was particularly acute in LTC settings, where workers often felt overwhelmed by high numbers of patients and the complexity of their needs. These challenges were seen as compromising the quality of care provided to patients and placing an unsustainable burden on care aids, exacerbating their experiences of moral distress. One participant stated, ‘The chronic underfunding of long-term care facilities means we are constantly understaffed and overworked, which takes a heavy toll on our mental health’ (*LTC, racialized woman* PID 2034). Another stated, ‘My experience with moral distress stems mostly from our staffing levels and not having the time or capacity to take the time I needed with high-need/high-risk clients. It feels so disheartening going home at the end of the day feeling like you could have done more but didn’t have the time or had to prioritize other work’ (*LTC, racialized woman* PID 491).

Respondents suggested strategies for addressing staffing shortages across the healthcare sector. First and foremost, they called for increased government investment in healthcare, particularly in underfunded areas like LTC. One respondent emphasized, ‘Investing in our workforce by increasing funding will not only improve patient care but also reduce the burnout and moral distress that come from working in an under-resourced environment’ (*nurse, white woman* PID 2298). In addition to increasing overall funding, respondents advocated for improved financial oversight and accountability to ensure that funds are used effectively and reached where they are most needed. An LTC nurse remarked, ‘Transparency in how funds are distributed and spent is crucial. We need mechanisms to ensure that the money is reaching the frontlines where it’s most needed’ (*racialized man,* 2567). Finally, respondents called for targeted funding initiatives that specifically address the mental health and well-being of healthcare workers. These initiatives could include the provision of mental health services, stress management programmes, and resilience training.

#### Greater resources for rural health

Respondents from rural communities reported significant disparities in access to essential resources, including medical supplies, staffing, and funding. These inequities often left healthcare workers in these areas feeling under-resourced and overwhelmed, as they struggled to provide care with limited tools and support. One respondent from a rural healthcare setting expressed frustration, stating, ‘We’re expected to provide the same level of care as urban centers, but with a fraction of the resources. It feels like we’re being set up to fail’ (*nurse, racialized woman*, 2785). This inequity not only impacted quality of care but also contributed to a sense of moral distress among healthcare workers who were aware of the disparities and effects on patient outcomes.

Respondents emphasized the need for systemic changes to address these disparities and ensure more equitable distribution of resources. They called for policies that recognize the unique challenges faced by rural communities and allocate resources accordingly. This included targeted funding for rural healthcare facilities, incentives for healthcare professionals to work in these areas, and investment in virtual care technologies to bridge the gap in access to care.

One participant suggested, ‘There needs to be a recognition that one-size-fits-all policies don’t work. We need tailored solutions that address the specific needs of different communities, especially those that have been historically under-resourced’ (*LTC, racialized gender minority* 2894).

#### Addressing racism

Though the survey did not specifically ask about racism, a number of respondents mentioned it in their qualitative response clearly linking discrimination to moral distress. For example, of the 431 respondents working in the LTC sector mentioned racism. Respondents highlighted how systemic racism within healthcare institutions manifests in both overt and covert ways, significantly impacting their professional lives and mental health. The intersection of race and gender was salient. For example, one participant observed, ‘Racism is still very much rampant in our society, and gendered norms continue to perpetuate stereotypes’ (*LTC, racialized woman* PID 1768). 768). Respondents also described microaggressions, particularly directed at racialized and women healthcare workers, as having a cumulative impact on the mental health and job satisfaction. A racialized woman working LTC remarked, ‘Experiencing these daily microaggressions adds up. It makes coming to work stressful and affects my ability to focus and perform my duties effectively’ (1899). This quote highlights how such incidents, such as dismissive comments, assumptions based on race, or exclusionary behaviours, can collectively contribute to a distressing work environment. In addition to microaggressions, some respondents reported more overt forms of racism from colleagues and supervisors. For instance, a *racialized woman physician* shared, ‘People often assume I am a nurse or assistant instead of a physician because I am a woman of color. It’s frustrating and undermines my professional identity’ (PID 1510). These experiences reflect not only individual biases but also systemic barriers that prevent racialized women from being recognized as equal professionals in their fields, further contributing to moral distress.

Respondents emphasized the need for comprehensive policies that explicitly address both race and gender discrimination within healthcare settings. One recommendation was implementing ‘mandatory training sessions on historical contexts and systemic racism to ensure all staff members are aware of the challenges faced by their colleagues and patients’ (*nurse, racialized woman* PID 2178). Beyond training, respondents advocated for creating spaces within healthcare institutions where issues related to diversity and inclusion could be openly discussed. One participant suggested, ‘Creating spaces for open dialogue about diversity can help address underlying issues and foster a more inclusive environment’ (*LTC, racialized woman* PID 1945). These forums would provide a safe environment for staff to share their experiences, seek support, and collaboratively develop strategies to address systemic inequities. Respondents further called for concrete institutional policies that support racial equity. This included the establishment of clear protocols for reporting and addressing incidents of racism and discrimination, as well as the integration of equity principles into all aspects of organizational decision-making. A *racialized woman* working in *LTC* articulated the need for systemic change: ‘Policies should not only focus on training but also on creating a culture that values diversity and actively works to dismantle systemic barriers’ (2245).

The importance of leadership in driving changes was also highlighted. Respondents stressed that organizational leaders must demonstrate a genuine commitment to equity and inclusion by actively participating in diversity initiatives and holding themselves and others accountable. A nurse explained, ‘We want to see an executive sponsor lead the charge, simply not on the manager level, as shifts in thinking and action must be spearheaded by those who have influence and the ability to drive change within the organization’ (*racialized woman* PID 222).

## Discussion

In applying the socio-ecological framework to analyse healthcare workers’ comments on moral distress and coping strategies, we aim to highlight the multiple contextual influences on moral distress and the need for interventions and policy changes across different levels. Respondents emphasized that moral distress is not solely an individual experience but is shaped by systemic, organizational, and interpersonal factors, which either exacerbate or alleviate the distress. Systemic issues, such as racism and staffing shortages, were frequently cited as factors that affect individual well-being. Individual-level strategies, such as self-care or therapy, were considered crucial for managing distress but were significantly influenced by organizational support.

The findings underscore the need for systemic, organizational, and interpersonal reforms to address factors like underfunding, racism, and administrative disengagement, which compound moral distress. The link between systemic issues and organizational practices is particularly evident in how institutional efforts to address systemic racism or improve resource allocation could impact interpersonal relationships at work. This aligns with research demonstrating that poorly resourced settings contribute to emotional and cognitive moral distress when workers are unable to provide the care they deem necessary (Bourgeault *et al.,* 2020; Hegarty *et al*., 2022). When organizations fail to provide resources like proper staffing or effective leadership, the burden of coping with moral distress falls disproportionately on individuals. Moreover, organizational policies – such as benefits for mental health care – can enable individual strategies like seeking therapy. Without these supports, personal strategies lose effectiveness (Wu *et al.,* 2021). Interestingly, while respondents called for systemic changes to address the root causes of moral distress, they did not specify system-level mitigation strategies, which may reflect a sense of powerlessness to access or demand such measures.

Interpersonal relationships were critical in mitigating moral distress, as supportive teams provided emotional validation and camaraderie, which helped buffer against negative impacts. Strong leadership was especially important in fostering a culture of psychological safety where workers felt empowered to express concerns and seek solutions. This highlights the interconnectedness of organizational and interpersonal factors; positive organizational environments foster better peer relationships, which in turn support individual coping strategies. Effective leadership can reduce feelings of powerlessness and frustration, which are core drivers of moral distress. Additionally, organizational initiatives like peer support programmes help reinforce solidarity and shared responsibility in navigating ethical challenges (Cyr *et al*., 2016).

Respondents’ experiences further reveal the compounded effects of discrimination and inequity within healthcare settings. These findings align with broader literature documenting the effects of intersectional discrimination on mental health and job satisfaction (Kirkbride *et al*., 2024). Research has shown that marginalization affects recruitment and retention, with both covert and overt prejudices manifesting during the application process, in workplace environments, and in access to equitable opportunities (Bourgeault *et al*., 2019; Baumann *et al*., 2021; Jefferies *et al*., 2022). These studies underscore the importance of considering the specific characteristics of the Canadian healthcare workforce when analysing the multifaceted nature of moral distress. However, there is still a lack of research that applies gender, intersectional, or other equity-based forms of analysis to moral distress (Murage *et al*., 2024). This significant gap highlights the need for more research that incorporates intersectional analysis to better understand and address nuanced experiences and drivers of moral distress in the healthcare workforce.

Based on these findings, we recommend concrete, evidence-based policy actions: (1) governments should increase healthcare funding to address staffing shortages, reducing burnout and distress (West *et al*., 2016); (2) healthcare organizations should mandate anti-racism training and equity-focused hiring practices, improving retention and reducing discrimination (Bourgeault *et al*., 2019); (3) leadership development programmes that foster psychological safety should be implemented at all levels (Edmondson and Lei, 2014); (4) peer support programmes and regular debriefings should be established to build team resilience (Whitehead *et al*., 2015; Cyr *et al*., 2016); and (5) institutions should publicly report on progress in equity initiatives, ensuring accountability (National Academies of Sciences, 2021; Kurtzman *et al*., 2022). Embedding these measures can address structural roots of moral distress and create healthier workplaces which would have downstream impacts including improved quality of care for patients (Aiken *et al*., 2002; West *et al*., 2016).

This study relied on self-reported data, which may be subject to recall bias or social desirability bias. Additionally, there is a high risk of selection bias due to the use of convenience sampling. This type of sampling also limits generalizability and may not reflect the experiences of all members. A significant limitation was the exclusion of healthcare workers who lacked English proficiency and thus may face unique structural barriers related to moral distress. Future research should aim to include a more diverse sample across different regions and healthcare systems to explore potential variations in experiences of moral distress. In the present study, the sample was predominantly composed of LTC aides, with comparatively smaller representation from nurses and physicians. Distinct and targeted research is therefore needed to more fully understand how moral distress is experienced and navigated by these professional groups. Moreover, this paper reports on qualitative data from two open-ended survey questions. While this provided valuable insights, a more detailed qualitative study using interviews or focus groups could offer a deeper understanding of the nuances of moral distress, particularly concerning intersectional identities such as race, gender, and profession. Future research should also explore longitudinal approaches to better capture the evolving nature of moral distress over time, especially in response to changes in healthcare systems and policies.

## Conclusion

This study highlights the multifactorial nature of moral distress among healthcare workers in British Columbia, Canada, with systemic, organizational, interpersonal, and individual factors all contributing significantly. While respondents identified certain individual coping strategies to manage their distress, these strategies often reflected a sense of powerlessness in addressing the contributors of moral distress, such as underfunding, and bureaucratic barriers. This finding underscores the need for systemic reforms that go beyond individual efforts and fosters supportive work environments through organizational change and effective leadership. Although higher-level redress remains more aspirational than commonly pursued, structural interventions are crucial to making a lasting impact. By addressing the drivers of moral distress at multiple levels, healthcare systems can better support the well-being of their workforce and improve the quality of patient care.

## Data Availability

Upon reasonable request.

## Code availability

Upon reasonable request.

## Table 1. Long description

The table presents data on 734 respondents categorized by profession, gender, and radicalized persons. It includes three professions: long-term care providers, physicians, and nurses. For long-term care providers (598 respondents), 37 men (6 percent), 532 women (89 percent), and 29 gender minorities (5 percent) are listed, with 19 men (51 percent), 489 women (92 percent), and 14 gender minorities (48 percent) being radicalized persons. For physicians (30 respondents), 9 men (30 percent), 10 women (33 percent), and 11 gender minorities (36 percent) are listed, with 7 men (77 percent), 10 women (100 percent), and 9 gender minorities (82 percent) being radicalized persons. For nurses (106 respondents), 15 men (14 percent), 75 women (71 percent), and 16 gender minorities (15 percent) are listed, with 12 men (80 percent), 68 women (91 percent), and 12 gender minorities (75 percent) being radicalized persons.

Navigate back to Table 1..

## Table 2. Long description

A table categorizing strategies for mitigating moral distress among healthcare workers across socio-ecological levels. The table is divided into four main themes: individual behaviors, interpersonal relationships, community/organizational factors, and societal/systemic policies. Each theme is further broken down into subthemes with corresponding suggested strategies. The individual level includes creative activities and therapeutic practices with strategies like protected time outside of work and investments in mindfulness training. The interpersonal level focuses on collegial relationships and family and friends, suggesting regular team meetings and fostering personal connections. The community/organizational level addresses workplace safety, regulations and bureaucracy, and administrative support, recommending proactive safety measures, streamlined processes, and improved communication. The societal/systemic level tackles addressing racism, staffing shortages, and resources for rural health, proposing mandatory trainings, increased investments, and policies specific for rural communities.

Navigate back to Table 2..
